# Supplementary material for: The evolution of skin pigmentation-associated variation in West Eurasia
Source: Proc Natl Acad Sci U S A. 2020 Dec 21;118(1):e2009227118. doi: 10.1073/pnas.2009227118 (PMC7817156; doi:10.1073/pnas.2009227118)
Supplement: Supplementary File [file pnas.2009227118.sapp.pdf]

Supplementary Information for The evolution of skin pigmentation associated variation in West Eurasia

Dan Ju, Iain Mathieson

Corresponding authors: Dan Ju, Iain Mathieson
Email: danju@pennmedicine.upenn.edu, mathi@pennmedicine.upenn.edu

This PDF file includes:

Figures S1 to S16
Table S1
Descriptions for Dataset S1

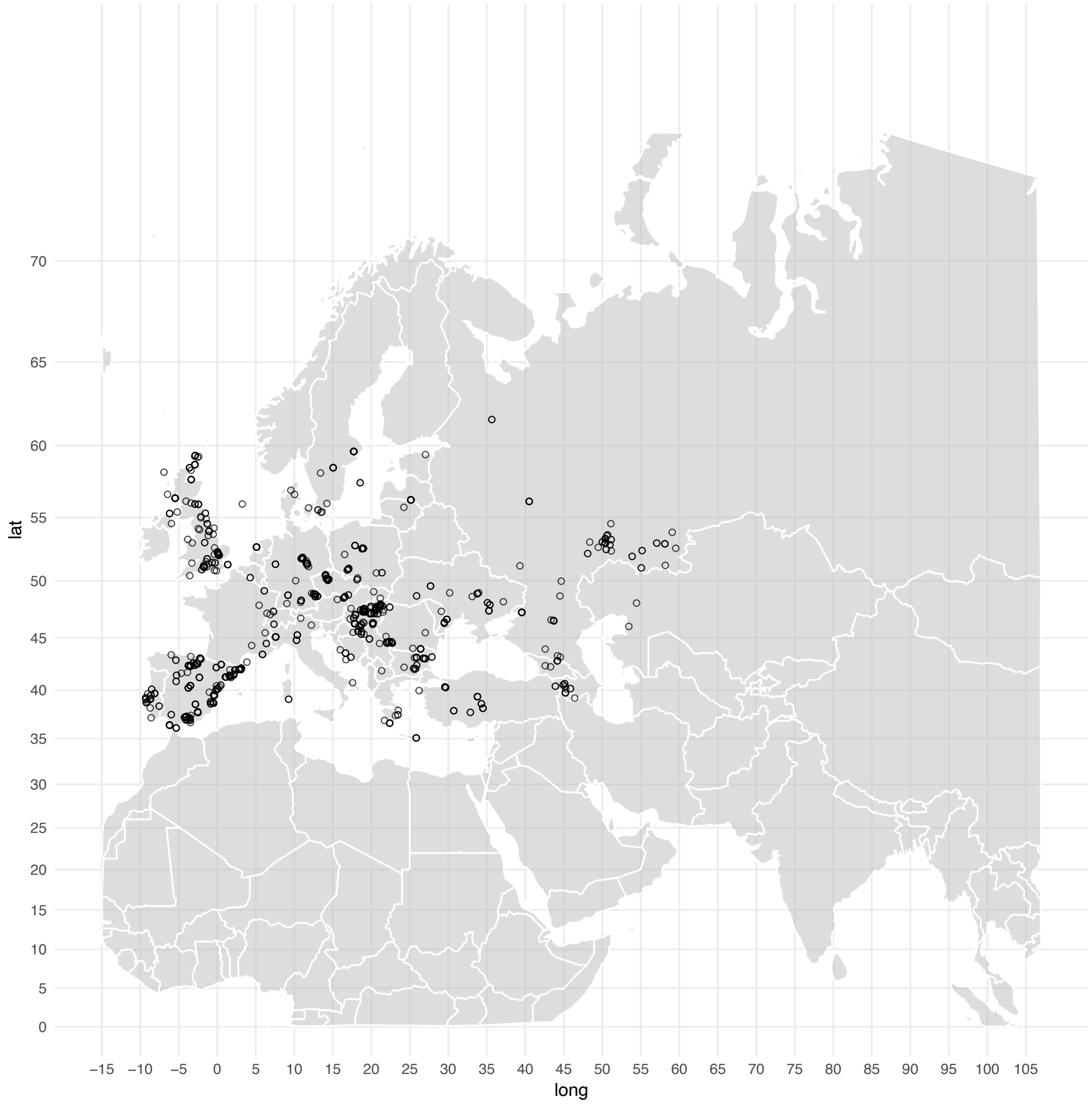

Fig. S1. Map of locations of ancient individuals included in the capture-shotgun dataset.

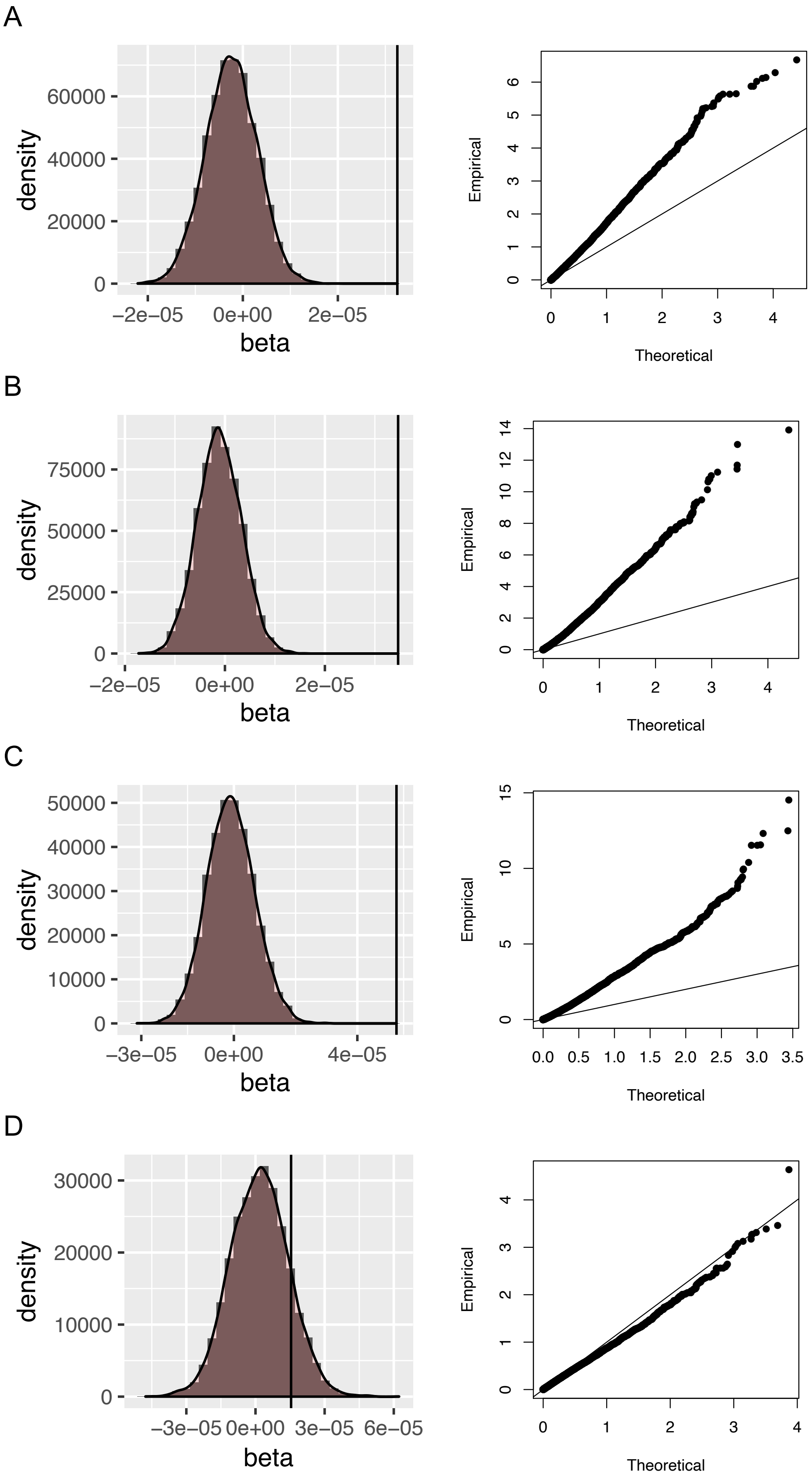
A
density
beta
60000
40000
20000
0
−2e−05
0e+00
2e−05
Empirical
Theoretical
B
density
beta
75000
50000
25000
0
−2e−05
0e+00
2e−05
Empirical
Theoretical
C
density
beta
50000
40000
30000
20000
10000
0
−3e−05
0e+00
4e−05
Empirical
Theoretical
D
density
beta
30000
20000
10000
0
−3e−05
0e+00
3e−05
6e−05
Empirical
Theoretical

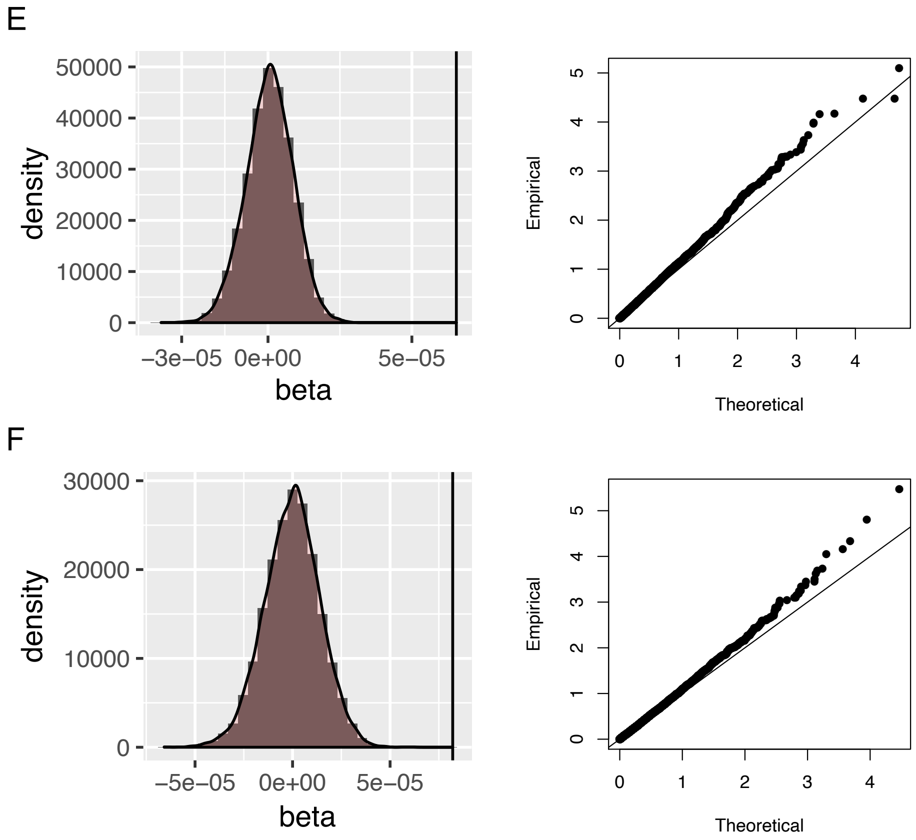

Fig. S2. Distribution of β_{date} from regression model for randomly generated score against time and Q-Q plot of -$\log_{10}$(P-value) of β_{date}. Randomly generated scores based on UK Biobank SNPs and manually curated SNPs were calculated for samples in the (A,D) shotgun dataset, (B,E) capture-shotgun dataset, and (C,F) capture-shotgun dataset dated within the past 15,000 years.

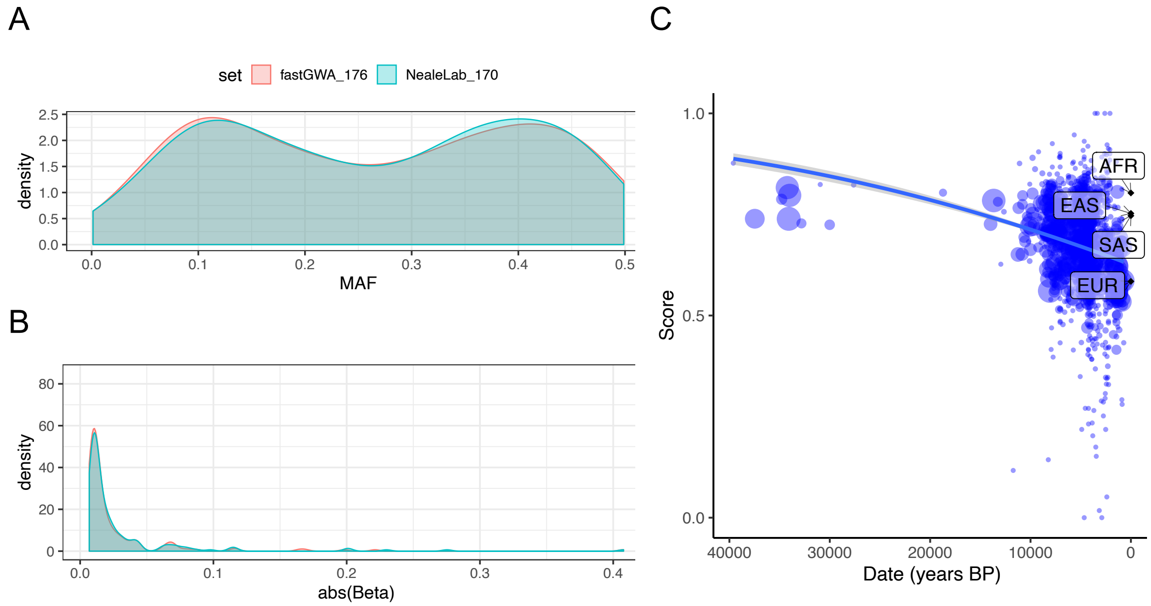

Fig. S3. Density plots of distributions of skin pigmentation associated SNPs from fastGWA (red) and Neale Lab (blue) for (A) minor allele frequency and (B) GWAS effect size. (C) Genetic score time series of SNPs from fastGWA in capture-shotgun dataset over 40,000 years ($P < 1x10^{-4}$).

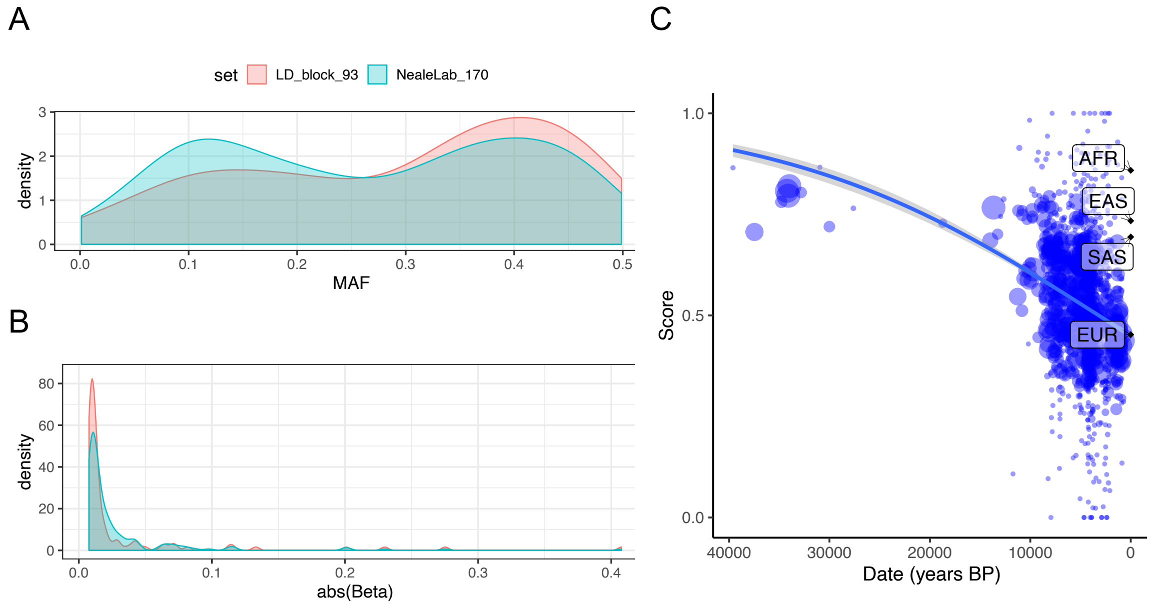

Fig. S4. Density plots of distributions of skin pigmentation associated SNPs from clumping on independent LD blocks identified from Berisa et al. 2016 (red) and clumping using PLINK (blue) for (A) minor allele frequency and (B) GWAS effect size. (C) Genetic score time series of SNPs from clumping using predefined LD blocks in capture-shotgun dataset over 40,000 years ($P <$ $1x10^{-4}$).

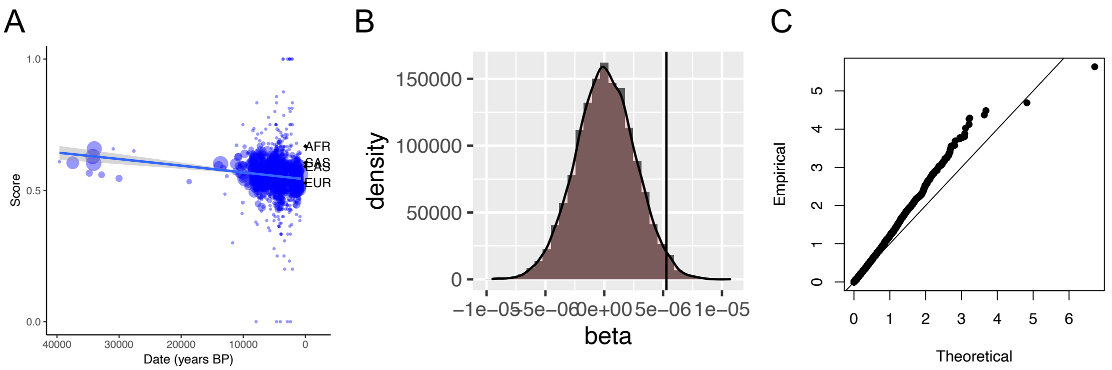

Fig. S5. (A) Regression of unweighted score based on 170 UK Biobank SNPs over time (*P* = 0.038). (B) Distribution of β_{date} from regression model for randomly generated score over time regressions and (C) corresponding Q-Q plot of -log$_{10}$(*P*-value).

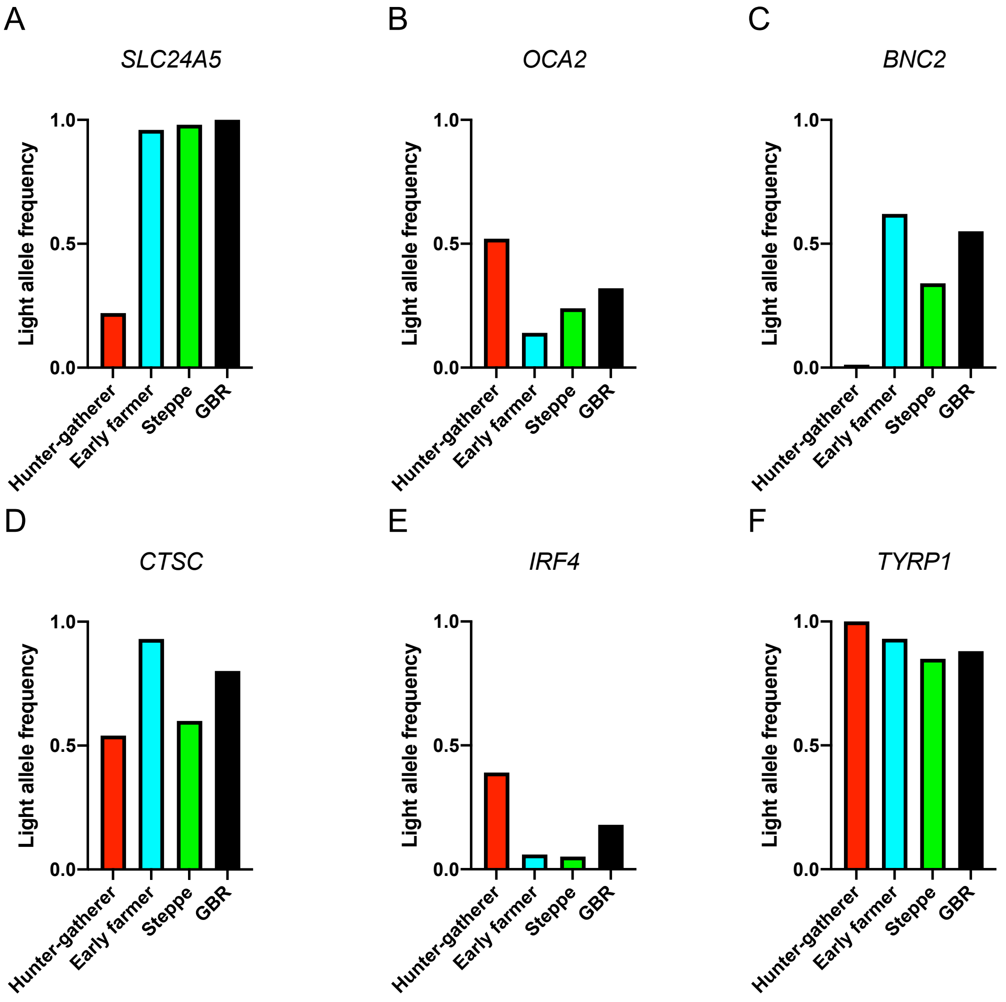

Fig. S6. Light allele frequencies in ancient and present-day European populations for select SNPs (A) rs2675345, (B) rs4778123, (C) rs2153271, (D) rs3758833, (E) rs12203592, and (F) rs1325132. Nearest genes are labelled at the top of each bar plot.

A

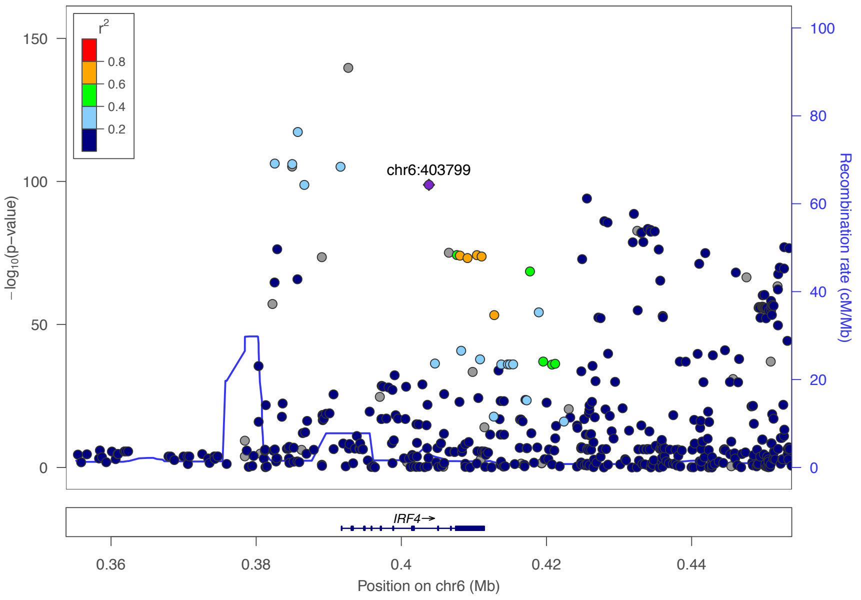

B

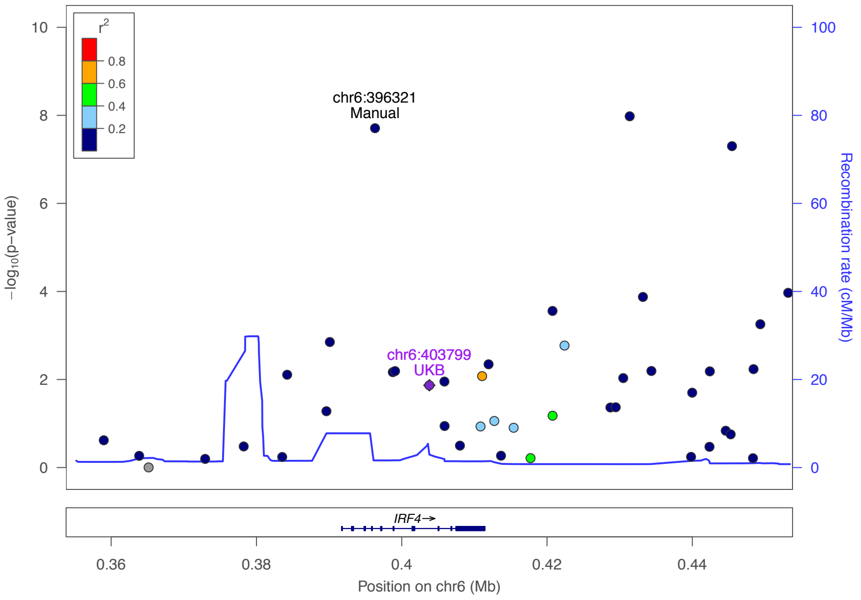

Fig. S7. (A) LocusZoom plot of the *IRF4* region for UK Biobank SNPs with the y-axis reporting P-values for the SNP association with skin colour association. Note: Low-confidence variants are included, but for SNP selection low-confidence variants were removed. (B) Plot of 1240K array SNPs at the *IRF4* locus with P-values corresponding to the ancestry term in the regression model for capture-shotgun 40,000 years with the manually curated SNP.

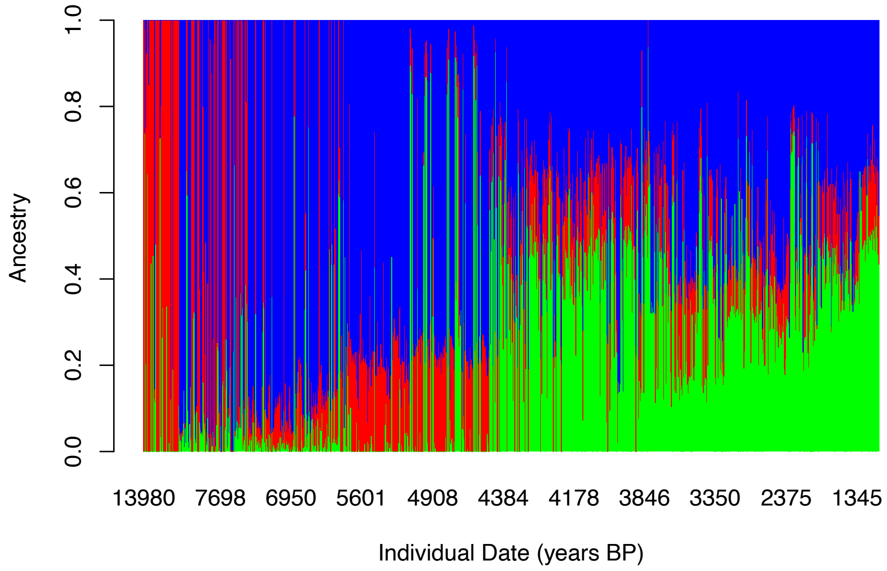

Fig. S8. Unsupervised admixture results (K=3) for all capture-shotgun ancient samples from 15,000 years BP onward.

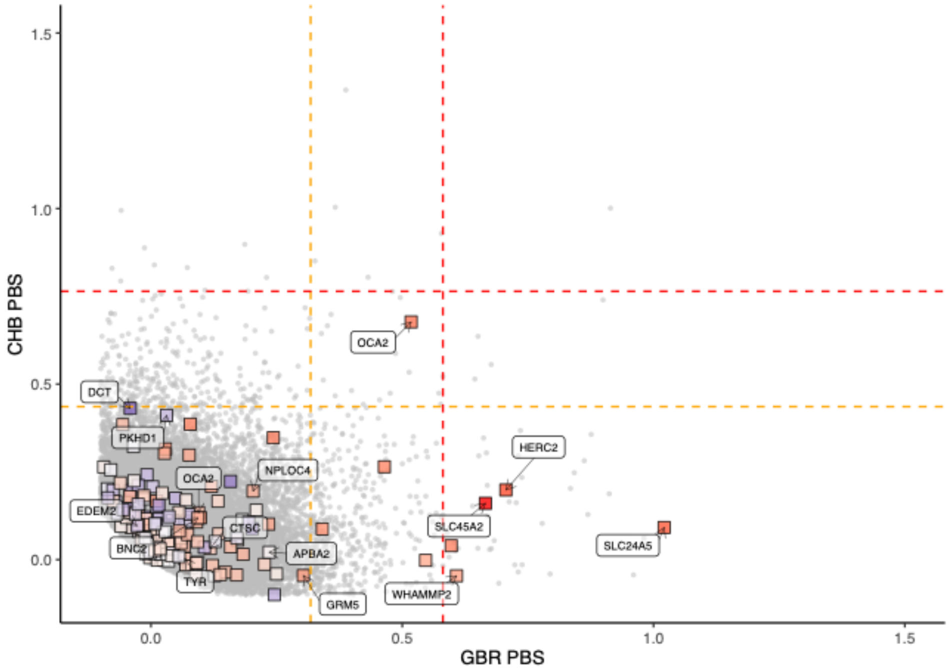

Fig. S9. Joint distribution of PBS for CHB and GBR from 1000 Genomes for the tree of GBR-CHB-YRI using 20 SNP windows. Boxes represent windows centered around a UK Biobank skin pigmentation SNP and are colored according to the magnitude of difference in frequency between GBR and YRI. Nearest genes are labelled. Orange and red lines represent top 1 and 0.1 percentiles.

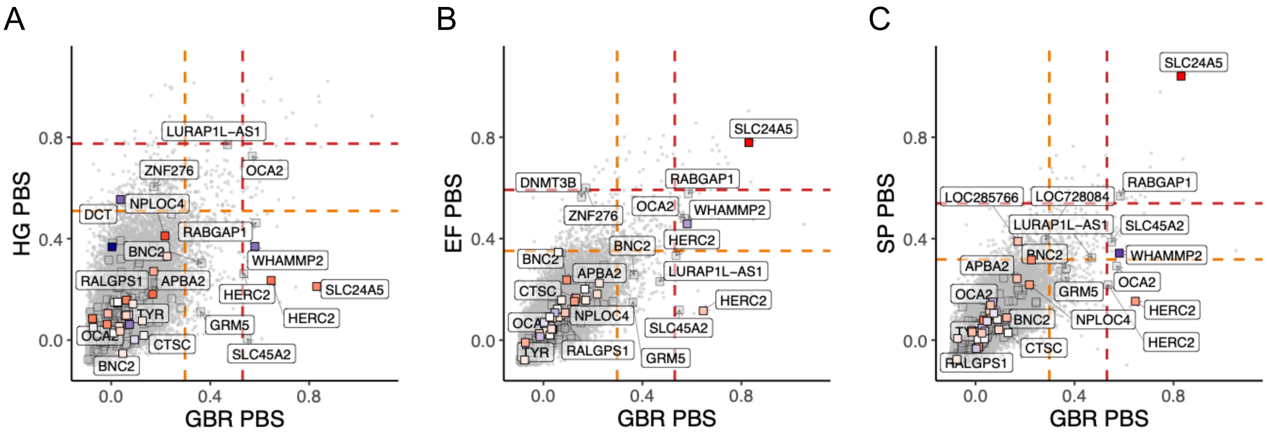

Fig. S10. Joint PBS distributions for non-overlapping 20 SNP-windows across the genome for GBR and (A) hunter-gatherer, (B) Early Farmer, (C) Steppe using YRI and CHB. Boxes represent windows centered around a UK Biobank skin pigmentation SNPs. Only windows containing SNPs at the extreme of the ancestry distribution from Fig. 2 are colored according to the magnitude of difference in frequency between the ancient group and YRI, whereas other windows are grey. Red indicates the light allele is higher in frequency in the ancient group. Nearest genes are labelled. Orange and red lines represent top 1 and 0.1 percentiles.

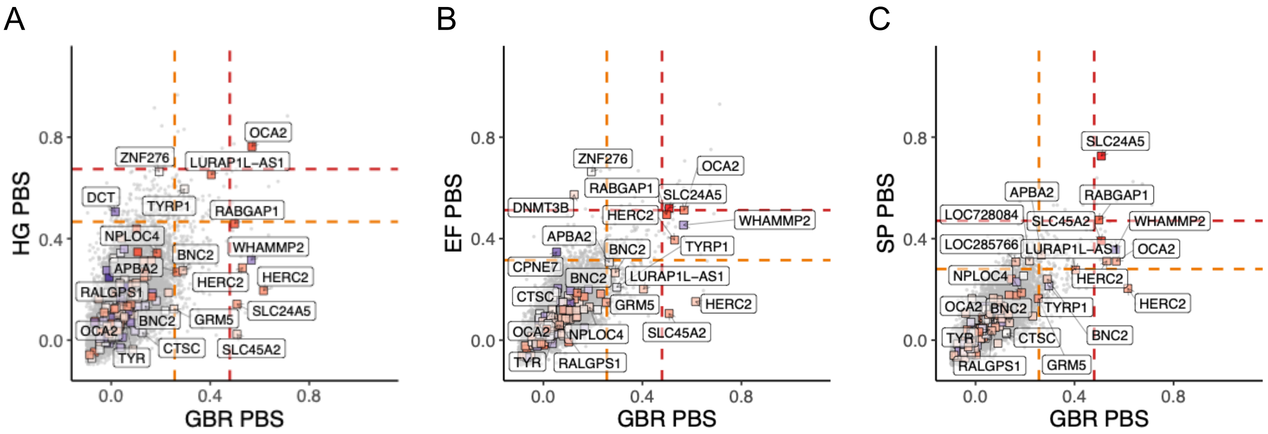

Fig. S11. Joint PBS distributions for non-overlapping 40 SNP-windows across the genome for GBR and (A) hunter-gatherer, (B) Early Farmer, (C) Steppe using YRI and CHB. Boxes represent windows centered around a UK Biobank skin pigmentation SNP and are colored according to the magnitude of difference in frequency between the ancient group and YRI. Red indicates the light allele is higher in frequency in the ancient group. Nearest genes are labelled. Orange and red lines represent top 1 and 0.1 percentiles.

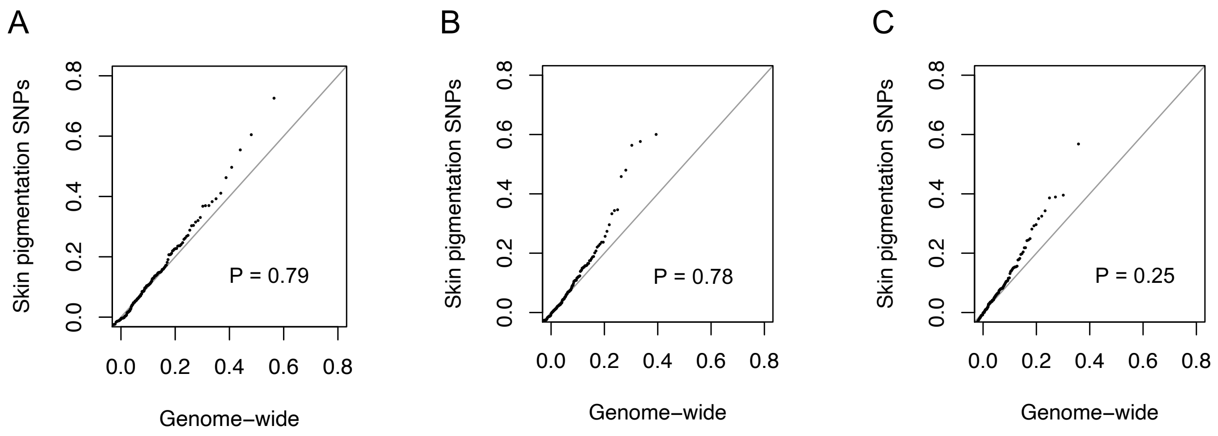

Fig. S12. Q-Q plots of distributions of PBS at skin pigmentation SNP-harboring genomic windows of 20 SNPs and PBS of all genomic windows for (A) hunter-gatherers, (B) Early Farmers, and (C) Steppe.

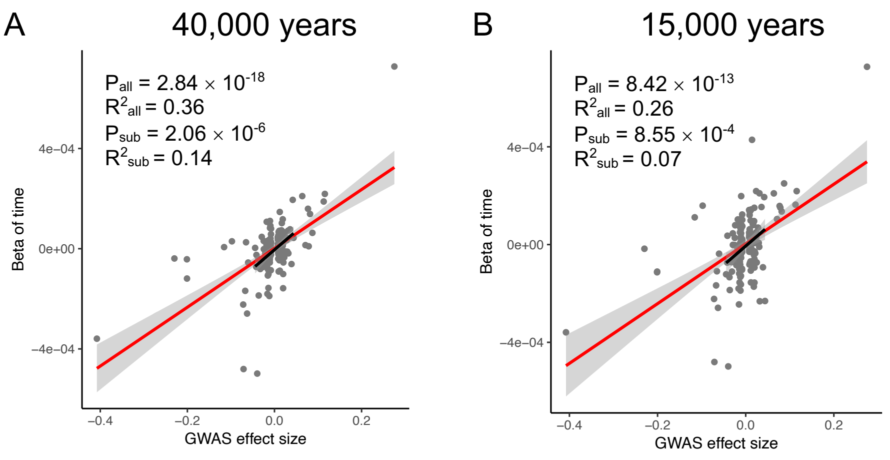

Fig. S13. Plots of 170 UK Biobank SNP GWAS effect size and magnitude of change in allele frequency represented by β_{date} in the full regression model for capture dataset over (A) 40,000 and (B) 15,000 years. Regression lines in red are based on 170 SNPs and statistics for this regression are denoted by "all," whereas the black line is based on 147 smaller GWAS effect size SNPs ($|\beta_{date}| < 0.05$) and statistics denoted by "sub."

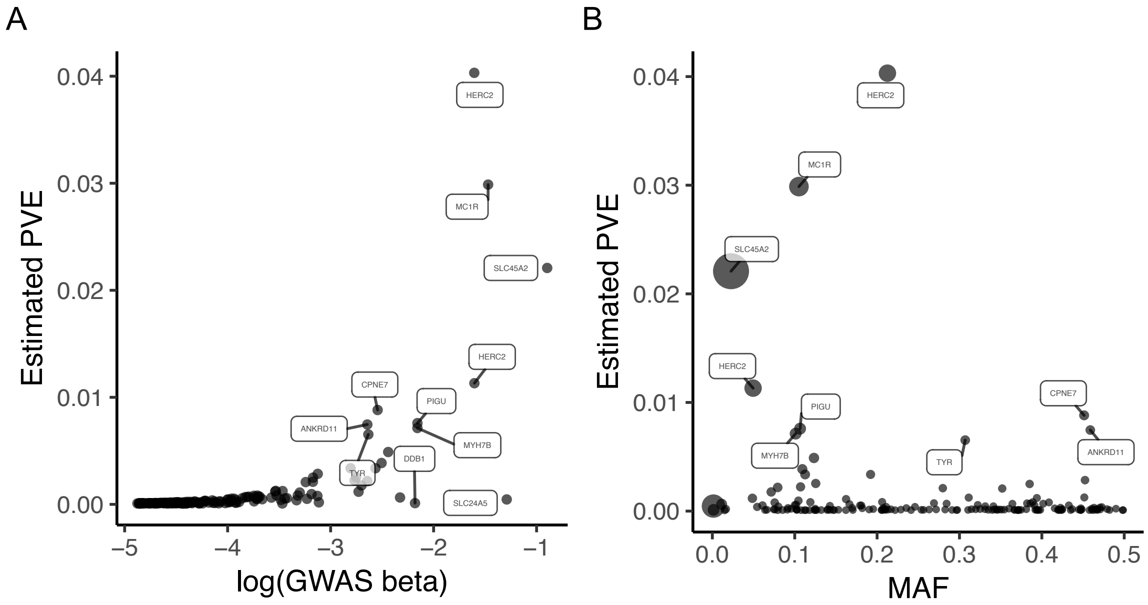

Fig. S14. (A) Estimated proportion of variance explained in the UK Biobank European population over GWAS estimated effect size of the 170 skin pigmentation SNPs. (B) Estimated proportion of variance explained over minor allele frequency in the UK Biobank European population. Point areas are scaled by effect size.

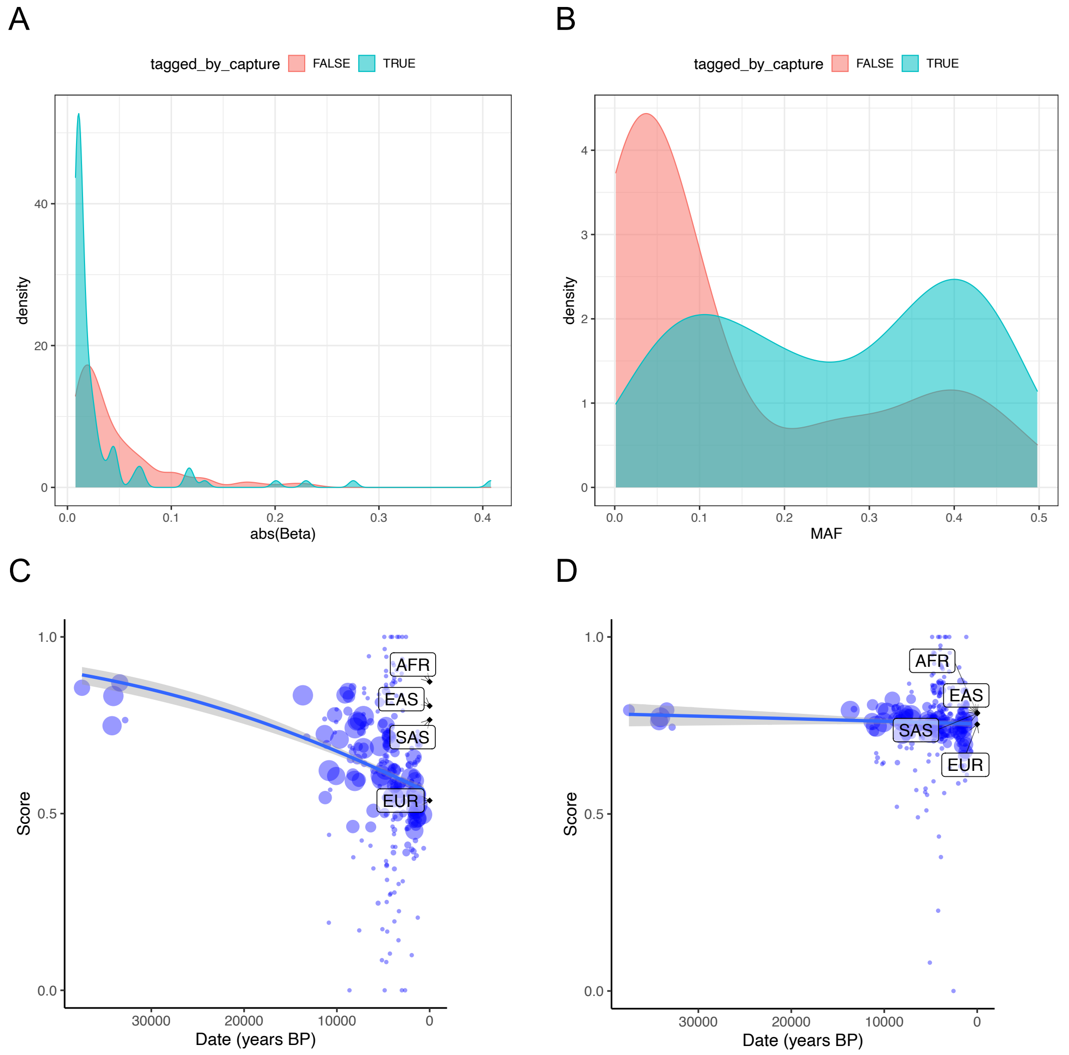

Fig. S15. Density plots of skin pigmentation associated SNPs tagged (at $r^2 \geq 0.8$) or not tagged by the 1240K capture array from Fig. 1A and 2A for (A) GWAS effect size and (B) minor allele frequency. Time series of genetic score for shotgun data based on (C) SNPs tagged by capture array and (D) SNPs not tagged by capture array. The difference between the present-day scores in present-day Europeans in subpanels C and D is because the SNPs that are not well-tagged have a higher average score. This is probably because the 1240K array is somewhat enriched for variants with signals of selection or functional effects.

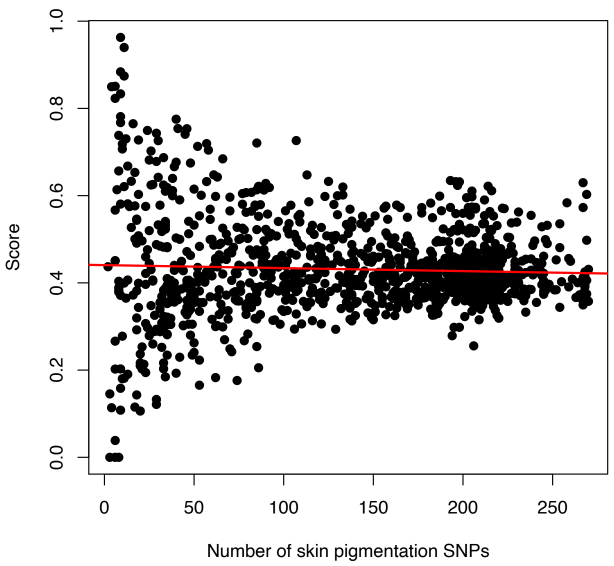

Fig. S16. Regression of skin pigmentation genetic score regressed on number of UK Biobank skin color SNPs present.

Name	UK Biobank SNP score	UK Biobank SNPs present	Manually curated SNP score	Manually curated SNPs present
Altai	0.81	169	0.67	18
Denisova	0.80	140	0.61	18
Les Cottés	0.86	59	0.71	17
Goyet	0.75	81	0.69	16
Mezmaiskaya 1	0.78	66	0.75	4
Mezmaiskaya 2	0.91	62	0.75	16
Spy	0.74	93	0.57	7
Vindija	0.84	153	0.67	18

Table S1. Weighted and unweighted genetic scores based on 170 UK Biobank SNPs and 18 manually curated SNPs, respectively, for Denisovan and Neanderthals.

Legends for Dataset S1 a-g

Dataset S1a. List of main set of 170 skin pigmentation-associated SNPs from the UK Biobank GWAS for skin colour conducted by the Neale Lab. These SNPs are all present on the 1240K capture array.

Dataset S1b. List of 242 skin pigmentation-associated SNPs ascertained from the Neale Lab, which were used in Figures 1A and 2A.

Dataset S1c. Variants implicated in skin pigmentation curated from the literature that were considered for the list of manually curated SNPs.

Dataset S1d. Publications considered for the manual curation of skin pigmentation SNPs.

Dataset S1e. List of 18 manually curated skin pigmentation-associated SNPs.

Dataset S1f. List of 93 skin pigmentation-associated SNPs from the Neale Lab based on predefined LD blocks from Berisa et al. 2016.

Dataset S1g. List of 176 skin pigmentation-associated SNPs from *fastGWA* conducted by Jiang et al. 2019.
